# Fast and low energy-consumption integrated Fourier-transform spectrometer based on thin-film lithium niobate

**DOI:** 10.1515/nanoph-2024-0219

**Published:** 2024-08-05

**Authors:** Xijie Wang, Ziliang Ruan, Kaixuan Chen, Gengxin Chen, Mai Wang, Bin Chen, Liu Liu

**Affiliations:** 12377 State Key Laboratory of Extreme Photonics and Instrumentation, College of Optical Science and Engineering, International Research Center for Advanced Photonics, Zhejiang University, Hangzhou 310058, China; Guangdong Provincial Key Laboratory of Optical Information Materials and Technology, South China Academy of Advanced Optoelectronics, Sci. Bldg. No. 5 & National Center for International Research on Green Optoelectronics, South China Normal University, Higher-Education Mega-Center, Guangzhou 510006, China; 12377 Jiaxing Key Laboratory of Photonic Sensing & Intelligent Imaging, Intelligent Optics & Photonics Research Center, Jiaxing Research Institute, Zhejiang University, Jiaxing 314000, China

**Keywords:** lithium niobate, Fourier-transform spectrometer, electro-optical modulator, spiral waveguide

## Abstract

Integrated miniature spectrometers have impacts in industry, agriculture, and aerospace applications due to their unique advantages in portability and energy consumption. Although existing on-chip spectrometers have achieved breakthroughs in key performance metrics, such as, a high resolution and a large bandwidth, their scanning speed and energy consumption still hinder practical applications of such devices. Here, a stationary Fourier transform spectrometer is introduced based on a Mach–Zehnder interferometer structure on thin-film lithium niobate. Long and low-loss spiral waveguides with electro-optic tuning are adopted as the optical path scanning elements with a half-wave voltage of 0.14 V. A high resolution of 2.1 nm and a spectral recovery with a bandwidth of 100 nm is demonstrated under a high-speed and high-voltage scanning in the range of −100 V to +100 V at up to 100 KHz. A low energy consumption in the μJ scale per scan is also achieved.

## 1 Introduction

Spectrometers are widely used in various scenarios such as light source calibration [1], chemical and biomedical analysis [2], agricultural monitoring [3], and satellite remote sensing [4]. Although desktop spectrometers based on discrete optical components can have an extremely high spectral resolution and a large bandwidth, their large size, expensive price, and sensitivity to vibrations limit their applications in portable devices [5]. So far, there have been many chip level spectrometers demonstrated on an integrated photonic platform. Classic light dispersive devices on a chip, such as array waveguide gratings [6], planar concave gratings [7], or array of resonators [8], can be used to split light in different wavelengths. In this case, the wavelength span of the spectrometer is limited by not only the number of spatial channels, but also the free-spectral-range (FSR) of the dispersive elements. Speckle based reconfigurable spectrometers using schemes of, e.g., multi-mode waveguide helix or resonant cavity modulation, can somehow overcome the constraint between the resolution and wavelength bandwidth [9], [10]. However, in order to achieve a large bandwidth, it is still necessary to include more channels and solve equations in a large scale, which leads to increased computational costs and time-consuming data processes [11]. The large number of detection channels would also limit the signal-to-noise ratio (SNR) of the spectrometer.

Fourier-transform (FT) spectrometer is another classic spectrometer structure, which can simultaneously detect an ultra-wide spectral bandwidth (which is inversely proportional to the small scanning step) and has a good SNR (the Fellget’s advantage) [12]. The main challenge for an on-chip FT spectrometer is how to achieve large optical path difference between the interference arms, since its resolution is directly determined by it. There have also been many demonstrations of such FT spectrometers based on silicon photonics. A spatial heterodyne spectrometer (SHS) which can achieve a resolution of up to 40 pm [13]. However, the under sampling resulted from the discrete spatial interference limits the spectral bandwidth to only about 0.75 nm. There is also a type of FT spectrometer that uses the thermo-optical (TO) effect of silicon to modulate the refractive index to achieve changes in optical paths [14]. This type of device only requires one Mach–Zehnder interferometer (MZI) structure to obtain the interferograms. A high resolution of 0.16 nm and a large bandwidth of 180 nm can be achieved using parallel structures [11]. However, the huge heating power up to about 9 W is required for achieving these performances, which is beyond the capability of a portable device. At the same time, the thermal expansion resulted from the elevated temperature and the nonlinear TO coefficient of silicon increase the complexity for the data processing. The schemes of using micro ring resonator (MRR) as a tunable filter to assist MZI or cascading MZI and SHS structures to break through the resolution of an FT spectrometer have also been proposed [15], [16], [17]. However, the slow response time of the thermal effect also limits the maximal scanning frequency to only about several Hz, which is unfavorable for fast spectrum sampling.

Lithium niobate, due to its high linear electro-optic (EO) coefficient of ∼30 pm/V, wide optical transparent window, and ultra-low optical loss, is widely used in commercial modulators. An MZI based miniature FT spectrometer has also been achieved based on lithium niobate using EO tuning [18]. However, due to the low modulation efficiency of bulk lithium niobate waveguide, a resolution of only 115 nm is achieved in the near-infrared band [19]. Recently, thin-film lithium niobate (TFLN) has emerged as a promising platform for integrated photonic devices [20], [21], [22]. The submicron sized waveguides on TFLN can have a high refractive index contrast, which helps further improve the modulation efficiency of EO modulators normally to 2.2 V cm or even less [23]. Its packaging size can also be reduced to about one tenth of traditional ones based on bulk lithium niobate. This is very attractive for integrated spectrometer chips. On the TFLN platform, a single interferometer has been used to generate complete interferograms between two counter-propagating waves for constructing a stationary-wave integrated Fourier transform spectrometer (SWIFTS). This device favors a large bandwidth of 500 nm and a moderate resolution of 5.5 nm [24]. By sampling asymmetric interferograms, the resolution can be improved to 1.2 nm [25]. However, a dense detector array is needed to capture the spatial interferograms, and only a small part of the standing-wave light is detected. This decreases the sensitivity and flexibility of the system. Alternatively, a standard MZI structure with 5.5 mm long modulation arms was fabricated for FT spectrometers on TFLN. This device exhibits a high half-wave voltage of 4.51 V, which limits its resolution to about 700 nm with a 20 V peak-to-peak scanning voltage [26].

In this paper, we demonstrate an MZI based stationary FT spectrometer on the TFLN platform. By using low-loss spiral waveguides for EO tuning, an ultra-low half-wave voltage of 0.14 V (*V*_π_) is obtained. The entire device size is 19 mm × 0.93 mm with an on-chip optical loss of only 5.5 dB. The scanning speed for obtain the interferograms can reach up to 100 KHz, thanks to the fast and linear EO response of lithium niobate. The peak power for scanning is about 5.90 mW at 1 KHz and an ultra-low energy consumption of 144 nJ is needed per scan. Using the standard FT procedure, the device presents a resolution of 2.1 nm and a bandwidth of 100 nm centered at 1,530 nm.

## 2 Structure and principle

**Figure 1:**
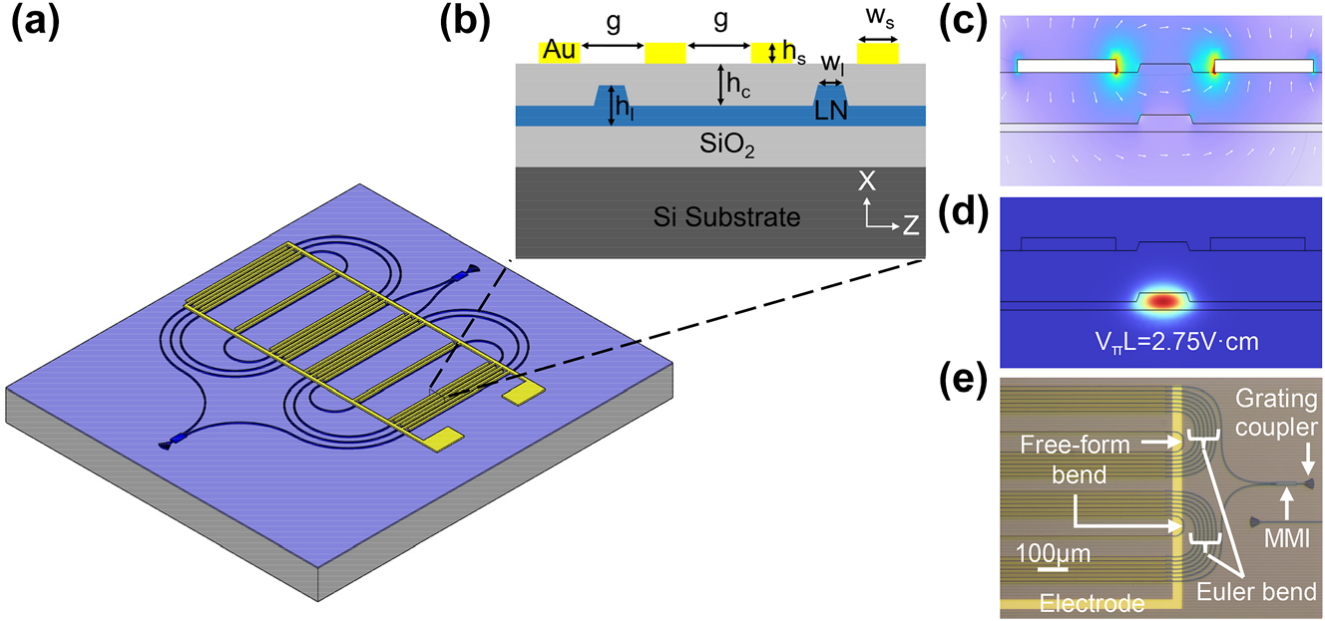
Proposed FT spectrometer based on the stationary EO tuning on the TFLN platform. (a) Schematic diagram of the whole structure. (b) Cross-sectional diagram of the tuning region. Simulated (c) electrostatic field distribution between the electrodes and (d) light field distribution of the fundamental TE mode. (e) Microscope image of part of a finished device.

The three-dimensional view of the TFLN based FT spectrometer is shown in Figure 1(a). Two multi-mode interferometers serve as the 3 dB couplers of the MZI structure. By applying a voltage to the lumped electrodes on the two spiral waveguides, the effective indices of the waveguide modes in the two arms are tuned to obtain the interferograms. The electrodes are arranged in a fashion that the induced changes in the effective indices are opposite, i.e., a push-pull configuration. The total length of each MZI arm is 27.5 cm. Figure 1(b) shows the cross-sectional view of the modulation section of the proposed FT spectrometer. The TFLN waveguide adopts a shallowly etched ridge waveguide structure with a width *w*_l_ of 1.5 μm, an initial TFLN thickness *h*_l_ of 400 nm, and an etching depth of 200 nm. In order to reduce the optical absorption loss caused by electrode misalignments, a SiO_2_ over-coating of thickness *h*_c_ of 1.2 μm and a relatively large electrode gap *g* of 3 μm is employed. The spacing between the spiral waveguides is 12 μm. The thickness of the electrode *h*_s_ is 300 nm. Based on the above structural parameters, the static electrical field and the optical field of the transverse electric fundamental mode (TE_0_) at this cross-section are shown in Figure 1(c) and (d). According to the simulation results, at 1,550 nm wavelength, the half-wave-voltage-length product is 2.75 V cm, and the theoretical optical waveguide loss due to metal absorption is negligible.

Due to the moderate refractive index contrast of TFLN structures, as compared to silicon-on-insulator for example, the bending radius of waveguides on the TFLN platform is generally large, if only circular arcs are used. The bending loss and cross-polarization coupling will increase largely if the bending radius is less than 100 μm [27]. However, a large bend will affect the compactness of the entire FT spectrometer based on spiral waveguide. Here, free-form bends are adopted as the central S-bends of the spiral, with a radius of only 30 μm [28]. Please refer to Supplementary Note 1 for passive waveguide structures, simulation and experimental results for transmission losses of the spiral waveguides.

The proposed spectrometer was manufactured on a commercial *x*-cut lithium-niobate-on-insulator wafer with a 3 μm thick buried oxide (BOX) layer and a 400 nm thick TFLN layer. The MZI structure was patterned using electron-beam lithography and inductively coupled plasma reactive ion etching. Then, plasma enhanced chemical vapor deposition was used to deposit a 1.2 μm thick silicon oxide over-cladding layer. 300 nm thick gold electrodes were prepared using contact photolithography, electron-beam evaporation, and lift-off processes. Due to the thick over-cladding layer, the alignment tolerance is released in the present structure, which is beneficial to ensure a very small optical absorption loss from the metal electrodes in the spiral waveguides. The image of a finished sample is shown in Figure 1(e).

## 3 Device calibration and spectrum recovery

**Figure 2:**
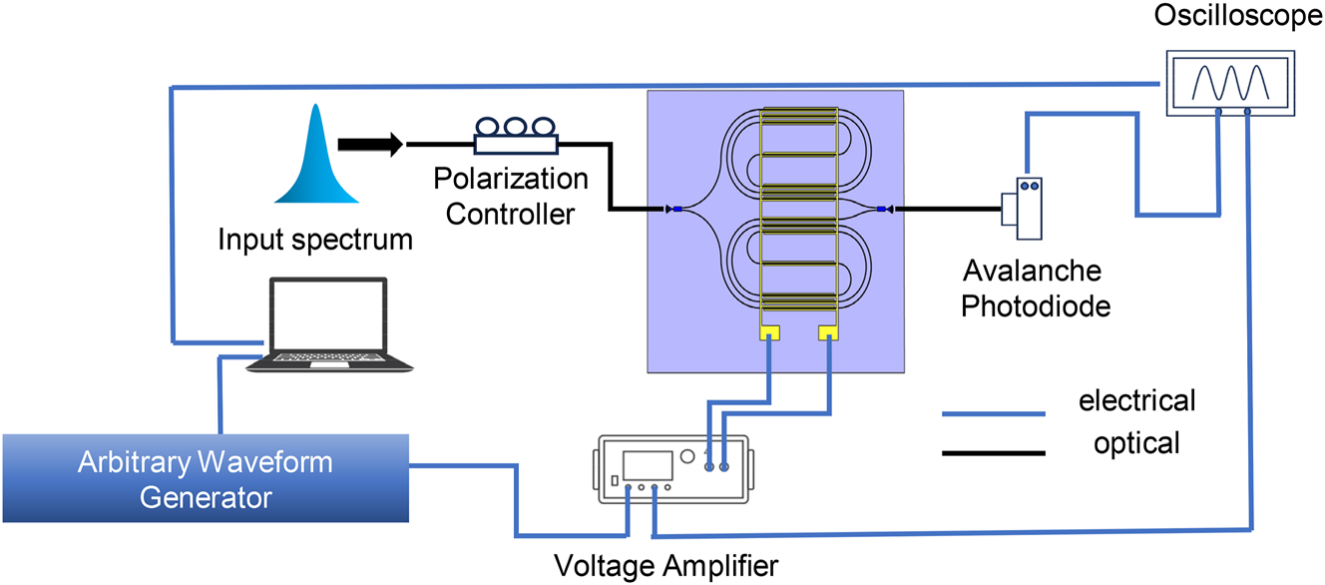
Experimental setup for capturing the interferograms from the proposed device.

The testing setup for the fabricated FT spectrometer chip is shown in Figure 2. Input light for spectral analysis is coupled into the device through a polarization controller and a focusing grating coupler [29]. The signal generator (20 MHz bandwidth) generated the scanning voltage, normally in a form of a triangular waveform. It was then amplified by a high-voltage amplifier (500 KHz bandwidth), and loaded onto the positive and negative electrode pads of the device. Thereby, the optical path difference between the two arms was modulated. The output interference signal was coupled out of the chip and sent to an avalanche photodiode (200 MHz bandwidth) again through a grating coupler. The detected signal, as well as the modulation voltage signal from the amplifier, was finally captured by an oscilloscope for further analyses. In order to prevent high-voltage breakdown of air, the chip surface was immersed in insulating silicone oil during measurements.

**Figure 3:**
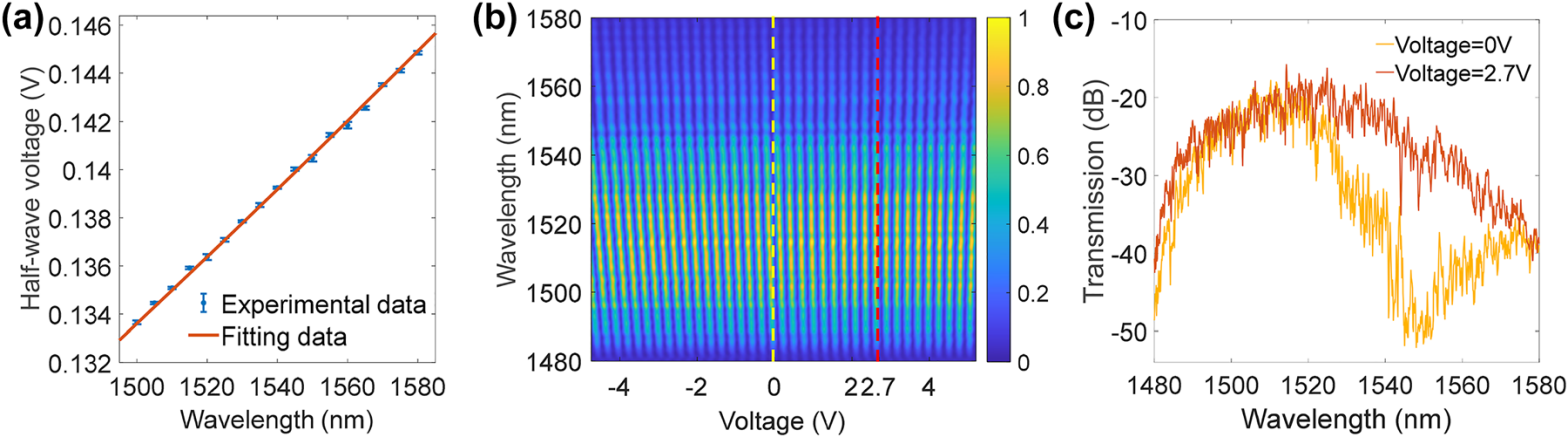
Calibration of the present FT spectrometer. (a) Relation between *V*_π_ and the wavelength. The experimental data was measured using a low voltage scan (±10 V) at each wavelength. (b) Voltage and wavelength mapping of the output response. (c) Measured transmissions of at zero voltage (yellow dashed line) and equal optical length with 2.7 V voltage (red dashed line), i.e., *T*(*λ*).

The calibration of the present FT spectrometer consists of two steps. First, the relationship between *V*_π_ and the wavelength should be measured. Since the wavelength information is embedded in, this relation is important for the wavelength accuracy of the reconstructed spectrum. The measured results are shown in Figure 3(a) with a wavelength interval of 5 nm. Keeping the first order approximation (see Supplementary Note 2), this curve here can be fit with the following equation:

(1) $$V_{\pi }\left(\lambda \right)=\frac{\lambda }{-4.049\lambda +17310}.$$

Here, *λ* is the wavelength in nm. Taking into account the modulation length of the spirals, i.e., the straight waveguides with the electrodes, 26.85 cm, the half-wave-voltage-length product of the fabricated device is about 3.76 V cm, which is slightly higher than the simulation result, probably due to the misalignment of the electrodes.

Second, the spectral response *T*(*λ*) of the present device itself should be deducted, which mainly includes the response of the grating couplers, the MMI splitters, and the spiral waveguides. Since *T*(*λ*) would be also embedded in the reconstructed spectrum, it is necessary to normalize it out for restoring the real spectral information of the input light. In the present device, a balanced MZI design is adopted. In principle, *T*(*λ*) can be measured directly from its transmission without any driving voltage. However, due to the long spiral waveguides and fabrication variations, the optical lengths of the two MZI arms are not equal, which can also lead to the device being modulated by an MZI envelope even at zero bias, as shown in Figure 3(c). To compensate this variation, a two-dimensional voltage-wavelength mapping of the device output was conducted as shown in Figure 3(b). Clearly, a grating-like pattern can be seen here, and the balanced point, where the optical length difference between the two arms is zero, can be drawn where the grating-line is parallel to the wavelength axis, i.e., at 2.7 V applied voltage for the present device. Therefore, at this point, the transmission spectrum of the device can be regarded as *T*(*λ*). As also shown in Figure 3(c), the device transmission includes the losses from the two grating couplers (∼12 dB) and the MZI loss (∼5.5 dB). From the two curves in Figure 3(c), one can also find that the extinction ratio of the present device is about 19.2 dB. Here, the wavelength range of *T*(*λ*) is mainly limited by the responses of the grating couplers in the present device. This can be relieved by using a broadband edge coupler instead [30], [31]. The oscillations of the transmission spectra mainly come from the scattering of spiral waveguides, and can be reduced by optimizing the fabrication technology.

**Figure 4:**
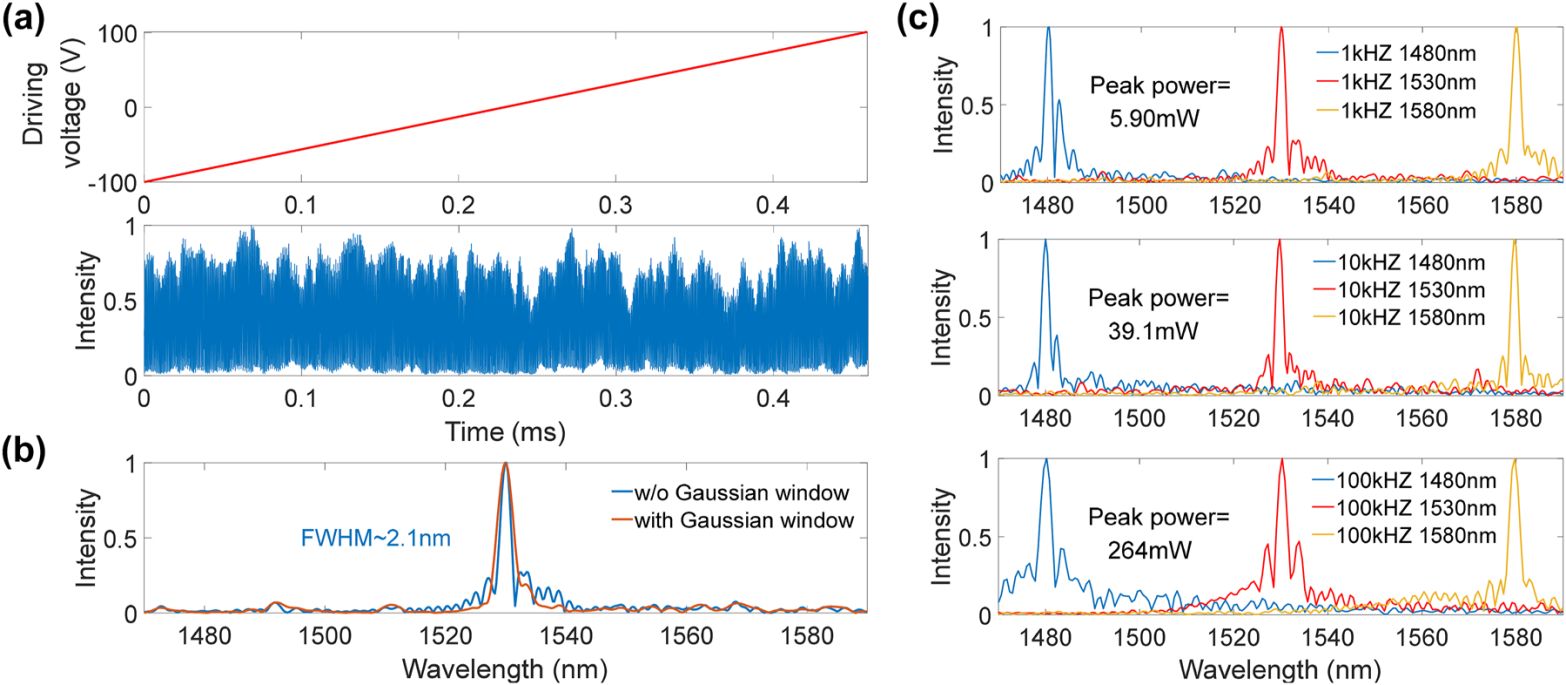
Spectrum recovery of a single wavelength laser. (a) Scanning voltage and the interferogram in time domain using a 1,530 nm single-wavelength input. (b) Corresponding recovered spectra with and without using a Gaussian window on the interferogram. (c) Recovered spectra of 1,480 nm, 1,530 nm, and 1,580 nm single-wavelength inputs at 1 KHz, 10 KHz, and 100 KHz scanning frequencies.

To characterize the performances of the present FT spectrometer, the spectral recovery ability of a single wavelength laser was first tested. A tunable laser source with an output power of 0 dBm at different wavelengths was brought to the chip. According to Figure 3(c), the peak power detected by the avalanche photodiode was about −17.5 dBm at 1,524.92 nm, and the average optical power between 1,480 nm and 1,580 nm was about −29.1 dBm. As described above, this loss is mainly due to the grating couplers and was effectively compensated with the receiving avalanche photodiode. Then, a triangular wave signal with 1 KHz frequency and 200 V peak-to-peak voltage (*V*_pp_) was applied to the device. The driving voltage and interferogram measured by the oscilloscope within one rising edge is shown in Figure 4(a), when the laser wavelength is at 1,530 nm. The total sampling time is only 0.5 ms in this case. The envelope oscillation of the interferogram mainly comes from the oscillations of the transmission spectrum as discussed above. Figure 4(b) shows the recovered spectrum through the FT and an appropriate coordinate transformation (see Supplementary Note 2), which exhibits a full-width at half-maximum (FWHM) of approximately 2.1 nm. The side-lobes of the recovered spectrum are resulted from the truncated interferogram due to the finite-voltage scanning. Using a Gaussian window can suppress these side-lobes with compromises in the resolution [32]. Theoretically, the resolution for a waveguide based FT spectrometer can be expressed as [33]:

(2) $$\Delta \lambda =\frac{0.603\lambda_{0}^{2}}{\Delta n_{g}L_{s}}=\frac{0.603\lambda_{0}^{2}}{\frac{V_{\text{pp}}}{4V_{\pi }\left(\lambda_{0}\right)}\cdot \frac{n_{\text{g}}}{n_{\text{eff}}}\cdot \lambda_{0}},$$

where, *λ*_0_ = 1,530 nm is the central wavelength, *n*_g_ = 2.221 and *n*_eff_ = 1.833 are the group and effective indices, respectively, of the optical mode at *λ*_0_. By taking into account Equation (1), Equation (2) gives Δ*λ* = 2.1 nm, which is consistent with the measurement results discussed above. Subsequently, the spectral recoveries at 1,480 nm, 1,530 nm, and 1,580 nm laser wavelengths were tested at scanning frequencies of 1 KHz, 10 KHz, and 100 KHz. As shown in Figure 4(c), the peak positions of all the recovered spectra match well with the corresponding input wavelengths. Due to the limited bandwidth of the voltage amplifier, the triangular-wave scanning voltage at 100 KHz only exhibits a linear range of *V*_pp_ = 140 V, which results in a decrease in the spectral resolution. Since a pure capacitive electrode structure is adopted in the present device, the electrical power consumption is anticipated low here, although the peak voltage is high in order to obtain a high wavelength resolution. By measuring the current flow during the scanning, we can quantitively examine the power consumption of the present device when capturing the interferograms (see Supplementary Note 3). At low frequency of 1 KHz, the peak power is about 5.90 mW, and the total energy consumption is 0.144 μJ per scan. As the scanning frequency increases, the peak power also increases. However, even under a high-speed scan at 100 KHz, the electrical power required by the present device is still orders of magnitude lower than that of the silicon-based FT spectrometers using TO tuning.

**Figure 5:**
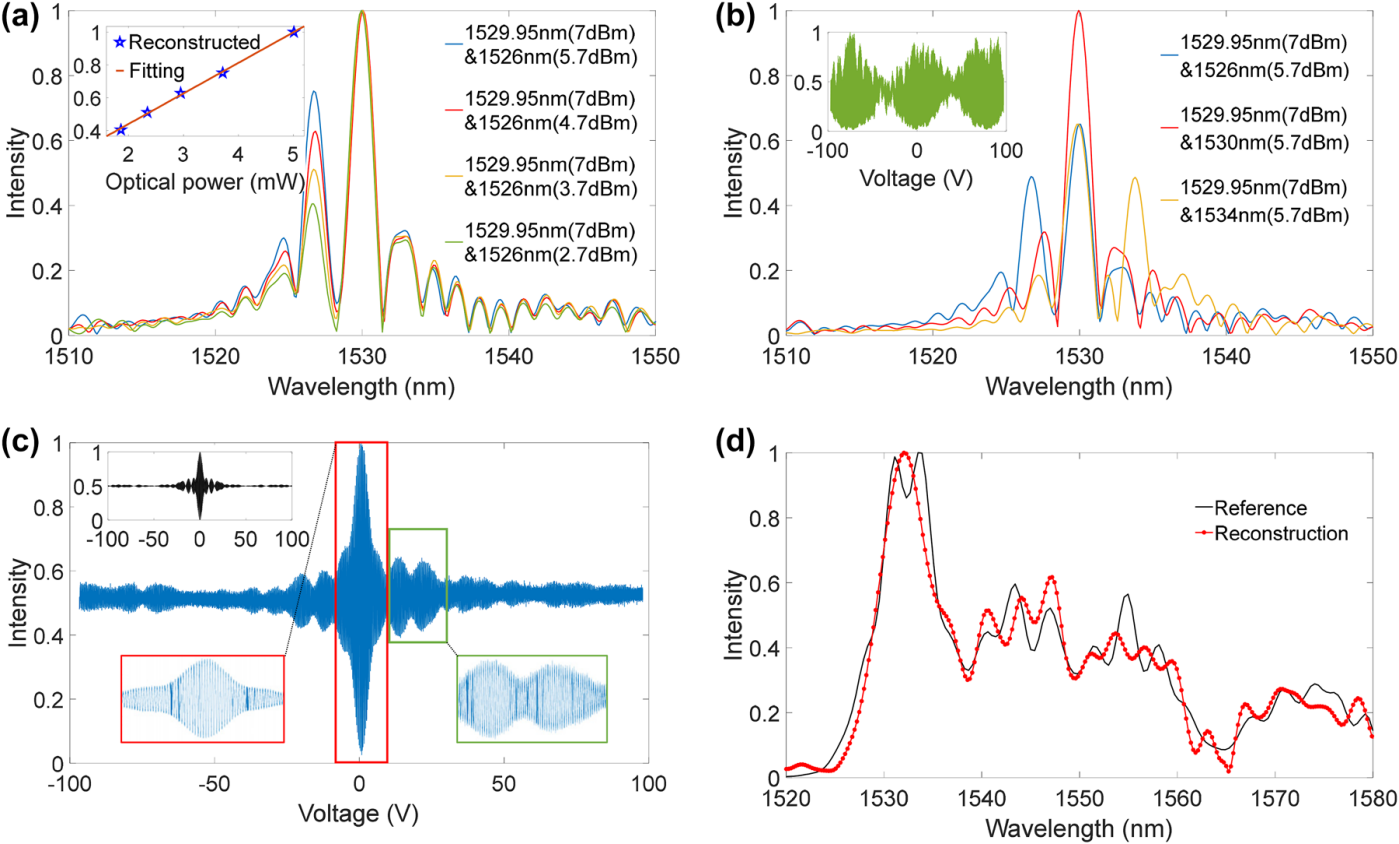
Dual laser and broadband spectrum recovery. Reconstructed spectra using dual laser inputs by changing (a) power of one laser and (b) wavelength of one laser. The inset of (a) shows the linear relationship between the recovered peaks and the actual laser powers. The inset of (b) shows the original interferogram with 1,529.95 nm and 1,534 nm laser input. (c) Measured interferogram with an ASE broadband light input. The black curve (inset) indicates the calculated interferogram, and the red and green boxes represent magnified views of the interferogram. (d) Recovered ASE spectrum (red curve) and the spectrum measured by a commercial OSA. Here, the transmission response of the device itself, i.e., *T*(*λ*), is normalized out.

Next, the responses of the present FT spectrometer to dual laser inputs with different powers and wavelengths were tested. Two tunable lasers after polarization controllers were combined and input to the chip. First, one laser was fixed at a wavelength of 1,529.95 nm and a power of 7 dBm, while the other laser had a wavelength of 1,526 nm and powers of 5.7 dBm, 4.7 dBm, 3.7 dBm, and 2.7 dBm. Figure 5(a) shows the recovered spectra, where the peak power at 1,529.95 nm is normalized. Since the difference of the two laser wavelengths is larger than the resolution of the present device, two extinguishable peaks can be seen in the recovered spectra. The recovered laser peak at 1,526 nm can also accurately reflect the variations of the input powers. There shows a good linear relationship between the recovered and actual input powers at 1,526 nm, as also shown in Figure 5(a). Second, while fixing one laser at 1,529.95 nm, we scanned the wavelength of the other laser at 1,526 nm, 1,530 nm, and 1,534 nm. Its power was fixed at 5.7 dBm. The recovered spectra in these cases are shown in Figure 5(b). Intuitively, two peaks are recovered in the spectra when the second laser is at 1,526 nm and 1,534 nm. For the case of 1,530 nm, the present device is not able to distinguish the two lasers, and a higher peak, which represents the sum of the two laser powers, presents around 1,530 nm. This confirms that the present FT spectrometer can achieve the spectral recovery of different wavelengths and power.

Finally, the spectral recovery performance of the device for an arbitrary broadband light was verified. An amplified spontaneous emission (ASE) light from an erbium doped fiber amplifier was input to the chip. The measured interferogram is shown in Figure 5(c). As a comparison, the theoretical calculated interferogram is also shown. One can find that measured interferogram shows correctly not only the main lobe around zero voltage but also the side lobes. The slight asymmetry of the interferogram is caused by the initial unequal optical lengths of the two arms as mentioned above [14]. The corresponding recovered spectrum is shown in Figure 5(d), together with the spectrum measured by a commercial optical spectrum analyzer (OSA). The two spectra are generally in good agreement in terms of the wavelength and overall shape. The small oscillations in the recovered spectrum may come from the 50-Hz power-line interference from the electronic devices and unshielded feed lines during the experiment, which can be relieved after chip packaging.

## 4 Discussion and conclusion

Table 1 summarizes the performance of some demonstrated on-chip FT spectrometers, including the present device. The rapid EO response of lithium niobate enables the scanning rate of the current FT spectrometer on the TFLN platforms to be increased by more than five orders of magnitude as compared to those on the SOI platform using the TO effect, while only requiring a low electrical power in the milliwatt range and a low energy consumption per scan in the μJ scale. As compared to the SWIFT structure [24], the standard MZI structure adopted here only requires one detector, which can be easily integrated onto the chip using hybrid integration. The scanning speed of the present device is limited by the bandwidth of the detector. At 100 KHz drive frequency, the frequency of the optical signal reaching the avalanche photo-diode was about 150 MHz, close to its bandwidth. Using a faster detector could further increase the scanning speed. The present device has proven that the EO-tuning based FT spectrometers can have a faster scanning speed than those based on TO or mechanical tuning.

**Table 1:** Comparison of some demonstrated FT spectrometers.

Platform	Type	Res. (nm)	BW. (nm)	Peak power (W)/voltage (V)	Energy per scan (J)	Time per scan (s)	Footprint (mm^2^)	No. of detectors
Si [14]	tFTS	3	1,522–1,578	5.1 W	∼2.55	∼0.5	1	1
Si [15]	tFTS + ring	0.47	1,526–1,616	1.8 W	69.16	11.4	0.2	1
Si [17]	DFT	0.2	1,550–1,570	>0.099 W	0.267	2.7	1.8	1
Si [11]	3MIsFTS	0.16	1,460–1,640	9 W	–	–	–	1
Si [16]	tFTS + SHS	0.25	1,450–1,650	2.4 W	0.06	0.025	33	128
TFLN [24]	SWIFTS	5.5	1,300–1,800	20 V	–	>0.002	10	87^a^
TFLN [25]	SWIFTS	1.2	1,310–1,635	15 V	–	–	20	89^a^
TFLN [26]	FTS	∼700	–	10 V	–	–	1.875	1
TFLN (this work)	FTS	2.1	1,480–1,580	0.0059 W/100 V	∼10^−6^	<10^−5^	17.67	1

^a^Number of scattering elements.

The resolution of the spectrometer is another key metric, and there is still room for improvement for the device structure demonstrated in this article. The maximal voltage that can be applied on the electrode is limited by the breakdown voltage of the SiO_2_ cladding, and theoretically can withstand more than 300 V. Additionally, due to the high-speed and low-energy consumption EO response of the present device, a high voltage impulse can be used for scanning. Such a voltage can be easily achieved with battery powered circuits. Due to the Jacquinot advantage of the FT spectrometer and the low-loss TFLN waveguide, it is possible to achieve sub-nm resolution using the proposed structure, by increasing the waveguide length and compromising the optical loss. By using micro ring resonators to assist the MZI structure, it is also possible to improve the resolution by orders of magnitude considering the high quality-factor ring that can be achieved on TFLN [15].

The footprint of the present device is also not fully optimized. The gaps between electrodes and between spiral waveguides were set large (3 μm and 12 μm, respectively). This helps release the process conditions in preparing the electrode patterns using contact photo-lithography. In the future, the spiral patterns can be designed compacter with narrower electrodes, and deep-ultra-violate stepper lithography can be used to define these fine patterns for both waveguides and electrodes. Besides, high modulation efficiency approaches, such as, slow-light waveguides [34] or Michelson structures using mirrors on the waveguides [35] can be adopted to further reduce the footprint of the device.

The wavelength bandwidth of the proposed device, from 1,480 nm to 1,580 nm, is limited by that of the grating coupler. Thanks to the Fellget’s advantage of the FT spectrometer, the edge bands within this range can still be accurately reconstructed. In order to further extend the wavelength range of the proposed structure, a broadband edge coupler, as well as a broadband beam splitter/combiner, on the TFLN platform can be adopted. Such devices have been demonstrated with a working wavelength range over 800 nm [31], [36], [37]. For spiral structures, compact waveguide bends covering a wide bandwidth also need to be considered [24], [28].

In summary, we introduced a high-resolution FT spectrometer chip on the TFLN platform. An MZI structure of a low half-wave voltage, 0.14 V, was achieved using low-loss spiral waveguides in the interference arms. The linear and fast EO effect of lithium niobate at a high voltage enabled not only a fast spectral recovery algorithm using only FT with a high resolution of 2.1 nm, but also a high scanning speed up to 100 KHz. Furthermore, the pure capacitive electrodes helped to maintain extremely low scanning power and energy consumption even at high scanning speeds. The present FT spectrometer structure could have potentials in applications of integrated biological and chemical sensing, hyperspectral imaging, and low-power remote sensing devices.

## Supporting Information

Further details about configuration of the FT spectrometer chip on TFLN; spectral recovery procedure; scanning power and energy consumption.

## Supplementary Material

Supplementary Material Details
