# Supplementary material for: Fast and low energy-consumption integrated Fourier-transform spectrometer based on thin-film lithium niobate
Source: Nanophotonics. 2024 Aug 5;13(21):3985–93. doi: 10.1515/nanoph-2024-0219 (PMC11501065; doi:10.1515/nanoph-2024-0219)
Supplement: Supplementary file 1 — Supplementary Material Details [file j_nanoph-2024-0219_suppl_001.pdf]

Supplemental Information for:

Fast and low energy-consumption integrated Fourier-transform spectrometer based on thin-film lithium niobate

Xijie Wang,[1] Ziliang Ruan,[1] Kaixuan Chen,[2,3] Gengxin Chen,[1] Mai Wang,[1] Bin Chen,[1] and Liu Liu[1,4]

[1]State Key Laboratory of Extreme Photonics and Instrumentation, College of Optical Science and Engineering, International Research Center for Advanced Photonics, Zhejiang University, Hangzhou 310058, China

[2]Guangdong Provincial Key Laboratory of Optical Information Materials and Technology, South China Academy of Advanced Optoelectronics, Sci. Bldg. No. 5, South China Normal University, Higher-Education Mega-Center, Guangzhou 510006, China

[3]National Center for International Research on Green Optoelectronics, South China Normal University, Guangzhou 510006, China

[4]Jiaxing Key Laboratory of Photonic Sensing & Intelligent Imaging, Intelligent Optics & Photonics Research Center, Jiaxing Research Institute, Zhejiang University, Jiaxing 314000, China

* Address correspondence to: liuliuopt@zju.edu.cn

Contents

1. Configuration of the FT spectrometer chip on TFLN
2. Spectral recovery procedure
3. Scanning power and energy consumption

Supplemental Note 1: Configuration of the FT spectrometer chip on TFLN

Figure S1 shows the waveguide structure of the present FT spectrometer chip. Its main part is the spiral waveguide arms with a total length of about 27.5cm each. The straight sections of length L_M=1.79cm are used for EO tuning, which gives a total modulation length in one arm of 26.85cm. Such a long modulation length makes the half-wave voltage of the present device extremely low. The spacing between the spiral waveguides is 12μm. Free-form bends of an equivalent radius R_F of 30μm are adopted in the central s-bends of the spiral, while Euler bends with an equivalent radius R_E of 66μm are adopted for the outer bending parts of the spiral.

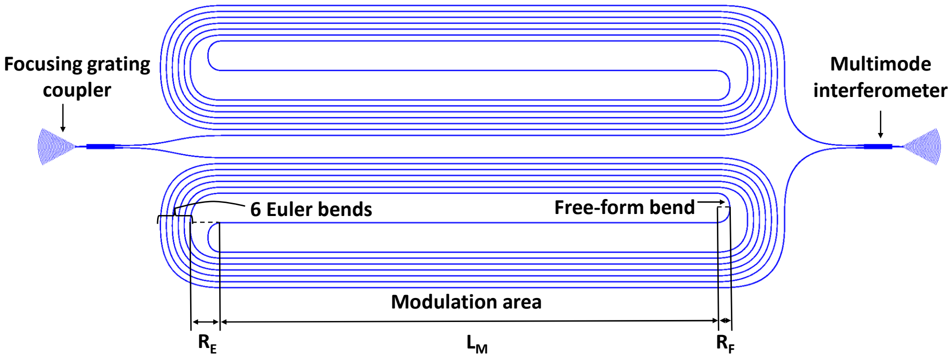

Figure S1. Schematic diagram of the waveguide structure of the proposed FT spectrometer.

Figures S2 shows simulated optical transmissions of a 90° free-form bend and a 90° Euler bend in the wavelength range of 1450nm to 1650nm, as well as their corresponding light propagations in the bends at the central wavelength of 1530nm. The 90° Euler bend exhibits an insertion loss less than 0.03dB [1], while the 90° free-form bend exhibits a higher insertion loss of 0.27dB, which is still acceptable considering the compact size of it.

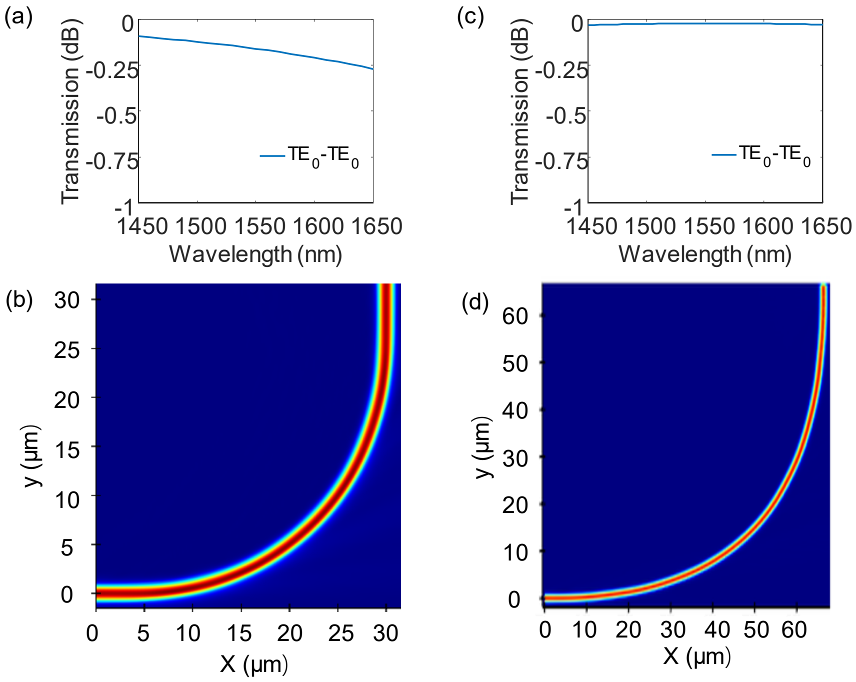

Figure S2. Calculated TE_0 transmissions and light propagations in (a & b) 90° free-form bend and (c & d) Euler bend. (b & d) are at the central wavelength of 1530 nm.

In the above structure, a ridge waveguide shown in Fig. 1(b) in the main text was adopted. The ridge width was set 1.5μm to minimize propagation losses. As shown in Fig. S3(a), this type of waveguide supports the presence of TE_0, TM_0, and TE_1 modes at the central wavelength of 1530nm. Additionally, from Fig. S3 (b & c), the 180° free-from bends and Euler bends in the spiral also serve as a filters for high-order modes. Therefore, high-order modes would be effectively suppressed in the present structure.

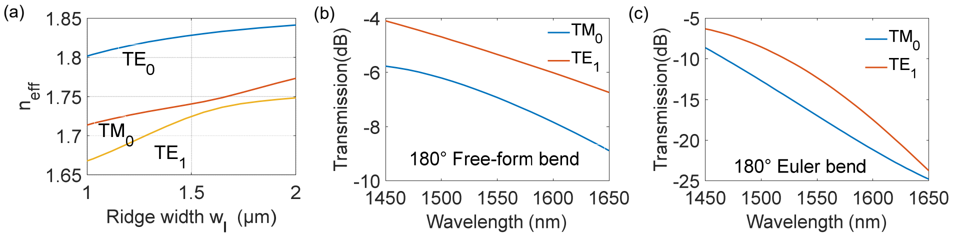

Figure S3. (a) Relations between the effective indices n_{eff} and the ridge width of the waveguide. (b) Light transmissions of (b) 180° Free-form bend and (c) 180° Euler bend for the TM_0 and TE_1 modes.

Transmission losses of spiral waveguides on TFLN are also evaluated with separate test structures with lengths of 10.29cm, 19.72cm, and 27.49cm. The measured transmissions of these test structures are shown in Figure S4. One can see that the propagation loss of the TFLN waveguide is about 0.1dB/cm-0.2dB/cm.

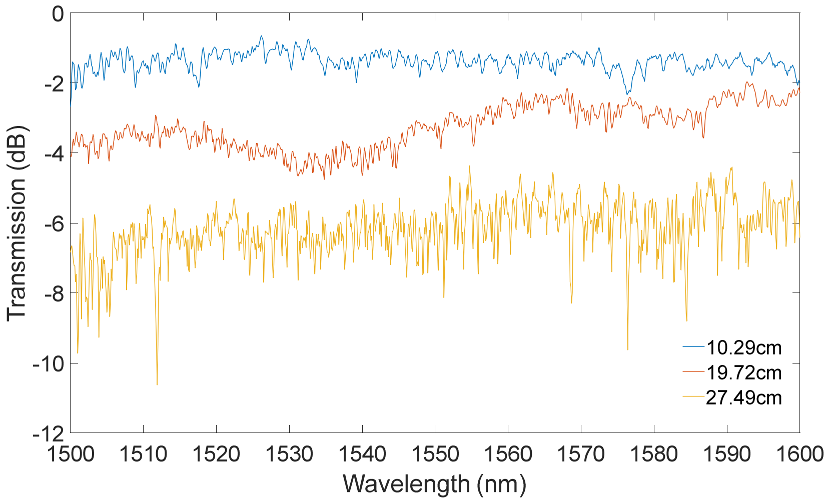

Figure S4. Transmissions of spiral waveguides on TFLN with different lengths.

Supplemental Note 2: Spectral recovery procedure

The working principle of the present FT spectrometer is based on the EO tuning of the MZI arms. A full numerical model was developed for calculating the static electrical field and optical mode field distributions in the waveguides as shown in Figures 1(c) and 1(d) in the main text. Considering the EO coefficients of lithium niobate material at different orders and the present device structure (maximal 100V voltage and ~3μm electrode gap) [2], only the linear EO effect (the Pockels effect) is considered for the change of the refractive index of the lithium niobate material in computational domain. Higher orders of the EO effects are therefore ignored. The optical path difference in the present MZI structure is determined by the variation of the mode effective index n_{eff}, which is a function of wavelength λ and applied voltage ΔV. We take its Taylor series expression (up to the third order) as:

$$\begin{aligned}\Delta n_{\mathrm{eff}}(\lambda, V) = &\left(\partial_V n_{\mathrm{eff}} + \partial_{\lambda,V} n_{\mathrm{eff}} \Delta\lambda + \frac{1}{2}\partial_{\lambda^2,V} n_{\mathrm{eff}} \Delta\lambda^2 + \frac{1}{6}\partial_{\lambda^3,V} n_{\mathrm{eff}} \Delta\lambda^3\right) V + \\ &\frac{1}{2}\left(\partial_{V^2} n_{\mathrm{eff}} + \partial_{\lambda,V^2} n_{\mathrm{eff}} \Delta\lambda + \frac{1}{2}\partial_{\lambda^2,V^2} n_{\mathrm{eff}} \Delta\lambda^2 + \frac{1}{6}\partial_{\lambda^3,V^2} n_{\mathrm{eff}} \Delta\lambda^3\right) V^2 + . \\ &\frac{1}{6}\left(\partial_{V^3} n_{\mathrm{eff}} + \partial_{\lambda,V^3} n_{\mathrm{eff}} \Delta\lambda + \frac{1}{2}\partial_{\lambda^2,V^3} n_{\mathrm{eff}} \Delta\lambda^2 + \frac{1}{6}\partial_{\lambda^3,V^3} n_{\mathrm{eff}} \Delta\lambda^3\right) V^3\end{aligned} \quad (S1)$$

where, $\Delta\lambda=\lambda-\lambda_0$ is the wavelength difference with respect to the central wavelength λ_0 of 1.53μm, V is the applied voltage on the electrode. $\partial n_{\mathrm{eff}}$ is the partial directive of the effective index along the subscripts at $\lambda=\lambda_0$ and V=0V.

Due to the push-pull structure adopted in the present device, the even-order EO effects, e.g., the second term of the right-hand side of Eq. (S1), are canceled out in the two arms. Therefore, only the odd-order terms need to be considered. We then define the following figures to compare different high-order terms in Eq. (S1) to the fundamental term [2]:

$$\xi_1 = \frac{\partial_{\lambda,V} n_{\mathrm{eff}} \Delta\lambda}{\partial_V n_{\mathrm{eff}}} \quad \xi_2 = \frac{\frac{1}{2}\partial_{\lambda^2,V} n_{\mathrm{eff}} \Delta\lambda^2}{\partial_V n_{\mathrm{eff}}} \quad \xi_3 = \frac{\frac{1}{6}\partial_{\lambda^3,V} n_{\mathrm{eff}} \Delta\lambda^3}{\partial_V n_{\mathrm{eff}}}$$

$$\chi_1 = \frac{\frac{1}{6}\partial_{V^3} n_{\mathrm{eff}} V^2}{\partial_V n_{\mathrm{eff}}} \quad \chi_2 = \frac{\frac{1}{6}\partial_{\lambda,V^3} n_{\mathrm{eff}} \Delta\lambda V^2}{\partial_V n_{\mathrm{eff}}}$$

$$\chi_3 = \frac{\frac{1}{12}\partial_{\lambda^2,V^3} n_{\mathrm{eff}} \Delta\lambda^2 V^2}{\partial_V n_{\mathrm{eff}}} \quad \chi_4 = \frac{\frac{1}{36}\partial_{\lambda^3,V^3} n_{\mathrm{eff}} \Delta\lambda^3 V^2}{\partial_V n_{\mathrm{eff}}}$$

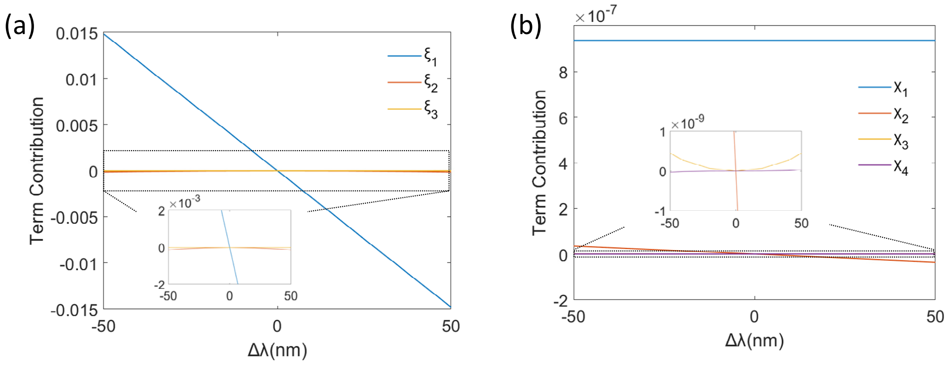

Figure S5. (a) Dispersion contributions of the first-order EO effects in the wavelength range of 100nm. (b) Ratios of third-order terms to the fundamental term in the voltage ranges of 200V.

According to Figure. S5, only ξ_1, i.e., the first-order dispersion, is significant in the current structure and parameter range. Therefore, the impact of higher-order effects could be ignored, and Eq. (S1) can be simplified as:

$$\Delta n_{\mathrm{eff}}(\Delta\lambda, V) = \left(\partial_V n_{\mathrm{eff}} + \partial_{\lambda,V} n_{\mathrm{eff}} \Delta\lambda\right)\Delta V. \tag{S2}$$

The experimentally measured interferograms $I(\Delta\phi)$ can then be expressed using the phase difference $\Delta\phi(\lambda)$ between the two arms of MZI as:

$$I(\Delta\phi) = \int_{-\infty}^{+\infty} S(\lambda) e^{j\Delta\phi(\lambda)} d\lambda, \tag{S3}$$

where $S(\lambda)$ is the power spectral density of the input light, which includes the transfer function of the MZI itself. Considering the push-pull structure and using Eq. (S2), the phase difference can be expressed:

$$\Delta\phi(\lambda) = \frac{2\pi}{\lambda}\left[2\Delta n_{\mathrm{eff}}(\lambda, V)L\right] = \frac{2\pi}{\lambda}\left[2\left(\partial_V n_{\mathrm{eff}} + \partial_{\lambda,V} n_{\mathrm{eff}}\lambda - \partial_{\lambda,V} n_{\mathrm{eff}}\lambda_0\right)VL\right], \tag{S4}$$

where *L* is the length of the modulation region of the spectrometer. The half wave voltage $V_\pi(\lambda)$ can then be defined as:

$$\frac{2\pi}{\lambda}\left[2\left(\partial_V n_{eff} + \partial_{\lambda,V} n_{eff}\lambda - \partial_{\lambda,V} n_{eff}\lambda_0\right)V_\pi(\lambda)L\right] = \frac{\pi}{2}. \tag{S5}$$

Generally, $V_\pi(\lambda)$ can then have the form of:

$$V_\pi(\lambda) = \frac{\lambda}{\alpha\lambda + \beta}. \tag{S6}$$

By fitting the experimental obtained V_π values shown in Figure 3(a) in the main text, $\alpha = -4.049\ \mathrm{V}^{-1}$ and $\beta = 17310\,\frac{\mathrm{nm}}{\mathrm{V}}$ can be obtained for the present structure. The phase difference can be simplified as:

$$\Delta\phi(\lambda) = 2\pi\left(\frac{V}{2V_\pi(\lambda)}\right) = 2\pi V g(\lambda), \tag{S7}$$

where $g(\lambda) = \frac{1}{2V_{\pi}(\lambda)}$ [3].

The interferogram can be rewritten as:

$$I(\Delta\phi) = I(V) = \int_{-\infty}^{+\infty} S(\lambda) e^{j2\pi V g(\lambda)} d\lambda = \int_{-\infty}^{+\infty} S(g) \frac{d\lambda}{dg} e^{j2\pi V g} dg = \mathrm{FT}\left\{S(g)\frac{d\lambda}{dg}\right\}, \tag{S8}$$

where FT{} is the Fourier transform. Then, the power spectral density of the input light can be recovered using the inverse Fourier transform of the above equation as:

$$S(g) = \frac{dg}{d\lambda} \mathrm{IFT}\{I(V)\} = \int_{-\frac{V_{\mathrm{pp}}}{2}}^{\frac{V_{\mathrm{pp}}}{2}} I(V) e^{-j2\pi V g(\lambda)} dV, \tag{S9}$$

where IFT{} is the inverse Fourier transform. Finally, using the coordinate transformation between *g* and *λ*, we can obtain the power spectral density of the input light $S(\lambda)$ in the wavelength domain:

$$S(g) \overset{g(\lambda)}{\rightarrow} S(\lambda). \tag{S10}$$

Supplemental Note 3: Scanning power and energy consumption

The measured voltage and current over time of the present FT spectrometer at scanning frequencies of 1KHz, 10KHz, and 100KHz are shown in Figures S6. Due to the bandwidth limitation of the detector, the scanning frequency up to 100KHz was tested. The energy consumption is estimated as the total electrical energy consumed in one scanning cycle. Here, the peak power is 5.90mW, 39.1mW and 264mW, and the total energy consumption is 0.144μJ, 0.578μJ and 1.00μJ respectively at scanning frequencies of 1KHz, 10KHz, and 100KHz.

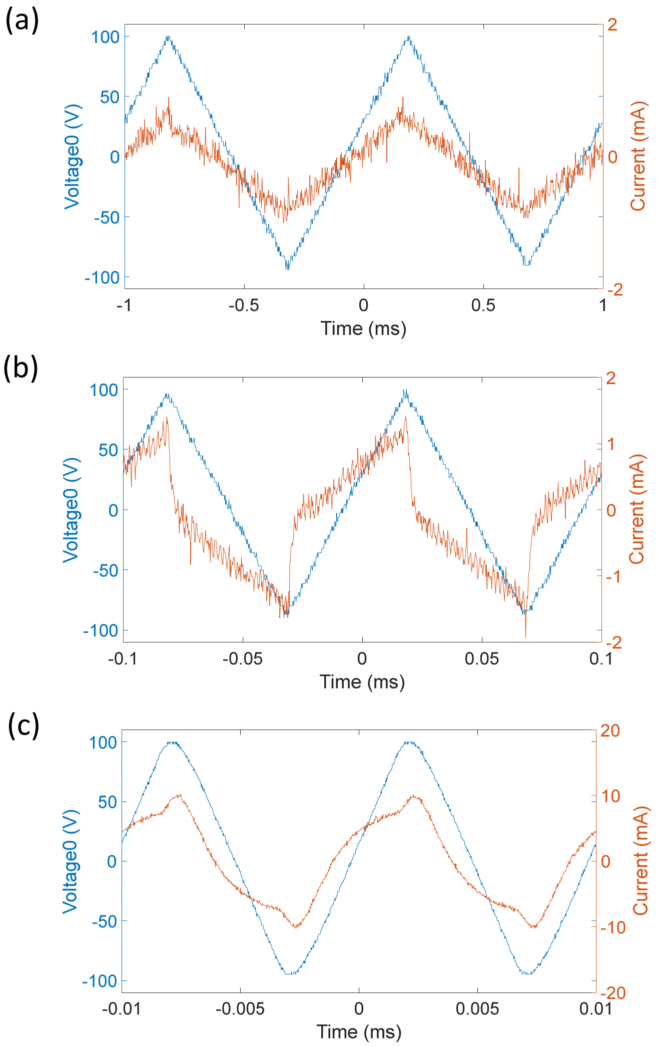

Figure S6. Measured voltage and current on the present FT spectrometer chip at (a) 1KHz, (b) 10KHz, and (c) 100KHz scanning frequencies.

REFERENCES

(1) X. Liu, Z. Ruan, S. Sun, et al., "Sharp bend and large FSR ring resonator based on the free-form curves on a thin-film lithium niobate platform," Opt. Express, vol. 32, no. 6, pp. 9433-9441, 2024.
(2) D. Zhu, L. Shao, M. Yu, et al. “Integrated Photonics on Thin-Film Lithium Niobate.” Advances in Optics and Photonics, vol. 13, no. 2, p. 242, 2021.
(3) L. Zhang, G. Gou, J. Chen, et al., “Miniature Fourier Transform Spectrometer Based on Thin-Film Lithium Niobate,” Micromachines, vol. 14, no. 2, 2023.
